# Mediastinal mixed germ cell tumor: A case report and literature review

**DOI:** 10.1515/med-2021-0293

**Published:** 2021-06-18

**Authors:** Xianwen Hu, Dandan Li, Jinhua Xia, Pan Wang, Jiong Cai

**Affiliations:** Department of Nuclear Medicine, Affiliated Hospital of Zunyi Medical University, Zunyi 563003, Guizhou Province, People’s Republic of China; Department of Obstetrics, Zunyi Hospital of Traditional Chinese Medicine, Zunyi 563003, Guizhou, People’s Republic of China; Department of Pathology, Affiliated Hospital of Zunyi Medical University, Zunyi 563003, Guizhou, People’s Republic of China; Department of Radiology, Weng’an Qingzhu Hospital, Weng’an 550400, Guizhou, People’s Republic of China

**Keywords:** mediastinal, mixed germ cell tumor, computed tomography, case report

## Abstract

Mixed germ cell tumor (MGCT) mainly occurs in young women’s ovaries and men’s testicles and rarely occurs outside the gonad. Fewer than 10 cases of mediastinal MGCT are available in PubMed, Embase, and other databases in English, while mediastinal MGCT with three pathological components, such as yolk sac tumor, immature teratoma, and embryonal carcinoma, has not been reported previously. A 12-year-old male sought medical attention for chest discomfort and underwent a computed tomography (CT) scan. A large soft tissue mass occupying most of the left thoracic cavity and mediastinum was detected. A CT-guided biopsy was performed, and an MGCT was diagnosed with pathological components, including yolk sac tumor, immature teratoma, and a small amount of embryonal carcinoma. Due to the large size of the tumor, the patient was treated with an EP regimen (etoposide + cisplatin) and paclitaxel + ifosfamide + cisplatin interstitial chemotherapy. The patient was followed up for 6 months and was alive with the disease. To the best of our knowledge, this is the 10th patient with MGCT in the mediastinum. The incidence of mediastinal MGCT is low, but it should still be considered one of the differential diagnoses of isolated pleural fibroma and neurogenic tumors.

## 1 Introduction

A mixed germ cell tumor (MGCT) is a relatively scarce tumor consisting of two or more germ cell components. It has a low degree of differentiation, and the most common combination is astrocytoma and yolk sac tumor (YST), derived from primitive germ cells of embryonic gonads [1,2]. Furthermore, MGCT mainly occurs in young women’s ovaries and men’s testicles and rarely outside the gonad, such as mediastinum, head and neck, and abdomen. Herein, we reported a case of mediastinal MGCT with pathological components consisting of YST and immature teratoma and a few embryonal carcinomas not been reported previously. We also reviewed the relevant literature, focusing on the CT and clinical characteristics of mediastinal MGCT.

## 2 Case report and literature review

### 2.1 Case presentation

**Figure 1:**
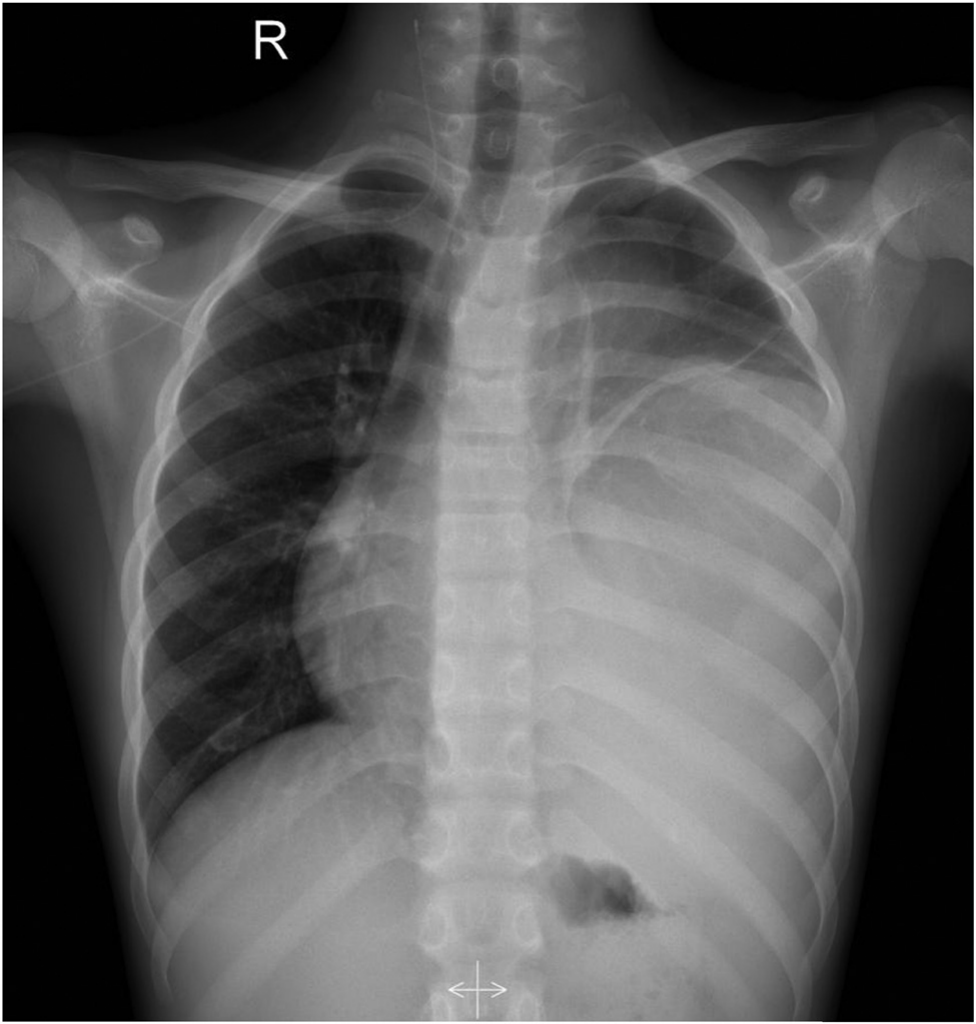
The chest X-ray showed a large dense shadow with an unclear boundary in the left lung field, and the heart was obviously compressed and shifted to the right.

**Figure 2:**
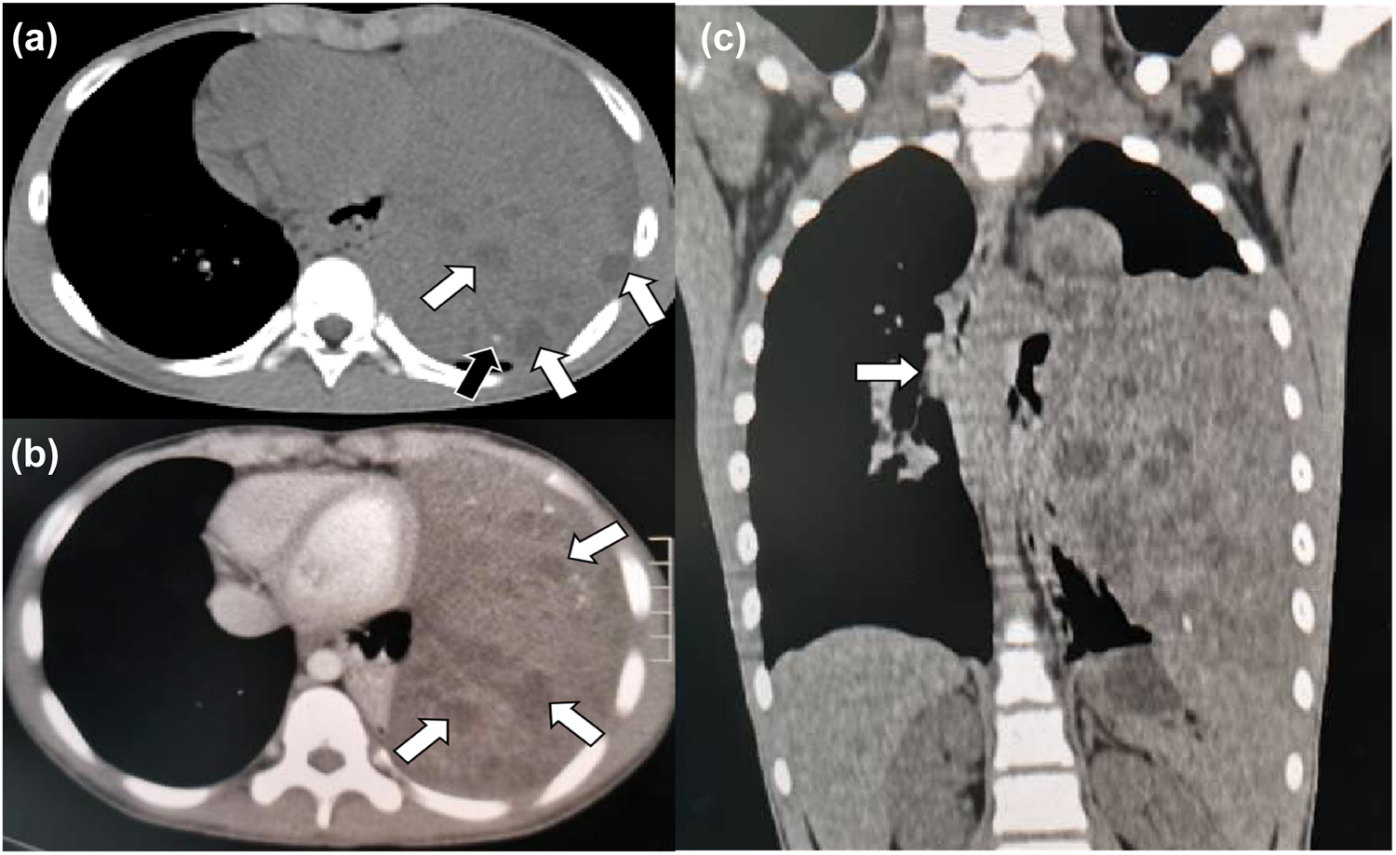
(a) Chest CT mediastinal window showed a huge soft-tissue density mass shadow in the left mediastinum, with unclear boundary, multiple nodular low-density areas (black arrow), and speckled high-density calcification foci (white arrow). (b) Contrast-enhanced CT revealed mild heterogeneous enhancement of the lesion, with nodular nonenhanced necrotic area (white arrow). (c) CT image of chest in coronal position. This coronal contrast-enhanced chest CT image clearly shows the extent of the mass, with the mediastinum slightly shifted to the right by the compression of the tumor (white arrow).

**Figure 3:**
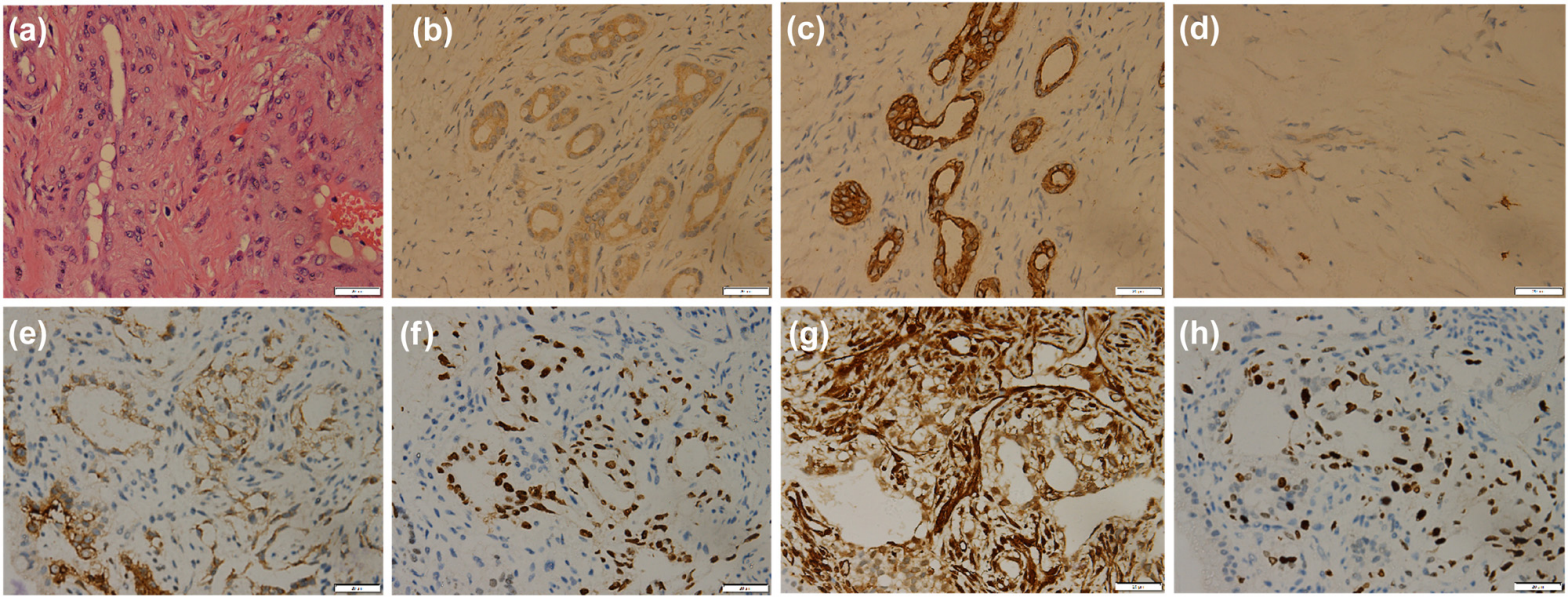
Hematoxylin-eosin staining (a), 40 times magnification: microscopically, there were obviously degenerate fusiform and ovoid tumor cells with loose reticular arrangement, hyperchromatic nuclei, inconsistent cell sizes, and can see the fat composition between the cells. Immunohistochemical staining showed tumor cells positive for AFP (b), CK (c), CD117 (d), glypican-3 (e), SALL4 (f), vimentin (g), and Ki-67 (h).


**Figure 4:**
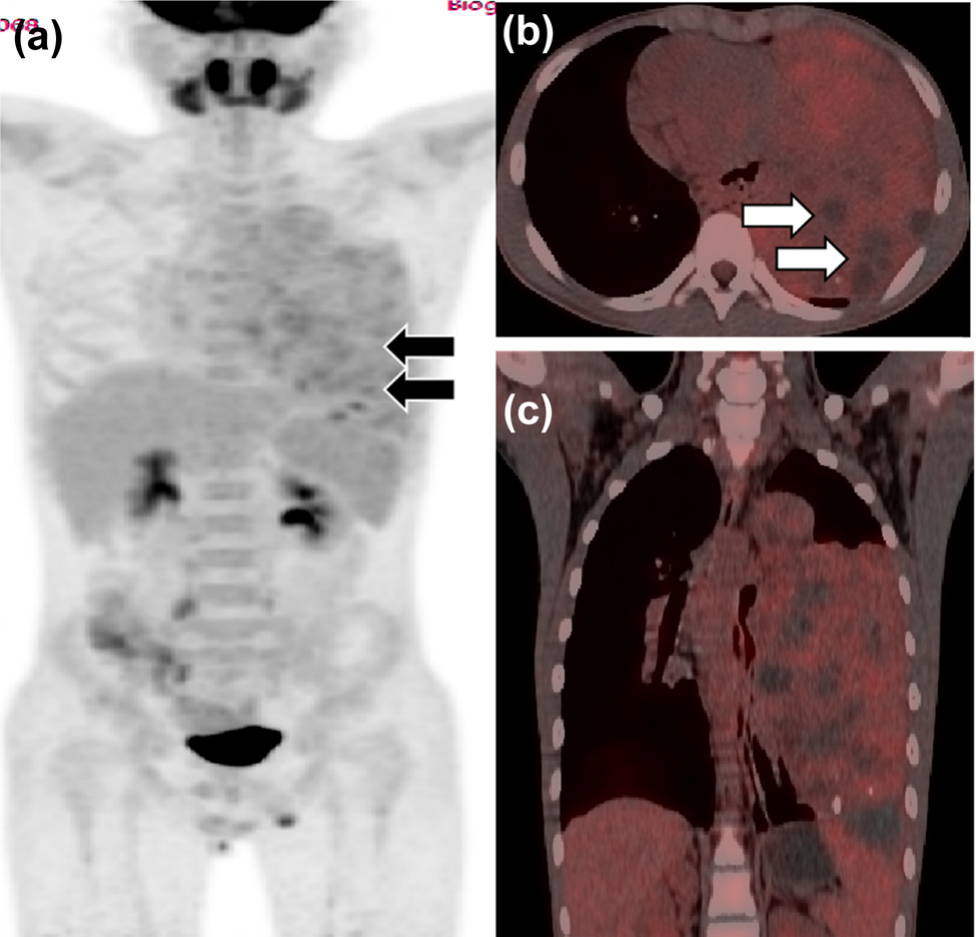
(a) PET/CT examination of the patient with mediastinal MGCT after chemotherapy; whole-body MIP (maximum density projection) images showed large patches of slightly increased radioactivity uptake in the left lung field (black arrow), SUVmax 5.2. (b) Axial PET/CT fusion images showed varying levels of uptake of radioactive tracers in the lesions, but no uptake in the cystic necrosis area (white arrow). (c) Coronal PET/CT fusion image.

A 12-year-old male was admitted to the hospital with cough, sputum, and fever for a week. The patient’s parents denied any history of family genetic diseases, tumors, infectious diseases, and any surgery. Physical examination revealed that the left lung breath sound was weakened, the percussion of the middle and lower lungs showed solid sound, and the body temperature was elevated to 38.3°C. Laboratory examination results showed that white blood cell (WBC) number and hemoglobin (Hb) level increased, and platelet (PLT), albumin, serum calcium, and phosphorus values decreased, while the remaining laboratory indicators were not abnormal. Tumor marker detection showed that neuron-specific enolase, carbohydrate antigen 199 (CA-199), 125 (CA-125), and alpha-fetoprotein (AFP) were significantly increased, and the results of the above laboratory tests and tumor markers are presented in Table 1. A chest X-ray plain examination revealed a large and dense shadow in the left lung field, and the heart was compressed and shifted to the right (Figure 1). The computed tomography (CT) image shows a large mass of soft tissue with uneven density in the mediastinum to the left thoracic cavity (Figure 2). The radiologist first considered it to be a single fibroma or neurogenic tumor. Based on the aforementioned examination results, the patient underwent CT-guided biopsy to confirm the diagnosis. The pathological and immunohistochemical images are shown in Figure 3. At high magnification, the tumor cells of different sizes are round or ovoid, arranged in loose meshes, and the endodermal sinus bodies can be seen between the cells. Immunohistochemistry showed that AFP, glypican-3, SALL4, CK, and vimentin were positive, CK5/6 squamous epithelium was positive, and OCT4 and CD117 were weakly positive. Moreover, CD34 was positive in tumor blood vessels, and Ki-67 was about 50% positive, while CD30, calretinin, MC, S100, and other tumor cells were not expressed. Based on these histopathological features, the pathologist diagnosed the tumor as an MGCT containing 75% YST and 20% immature teratoma, as well as 5% embryonal carcinoma. Due to the vast size of the tumor and its unclear boundaries with the heart and left large vessels, the patient could not undergo surgery and only received chemotherapy with VP16, cisplatin, cyclophosphamide, mesna, and bleomycin; cefotaxime sodium was used for anti-infective treatment. On day 5 posttreatment, the fever symptoms improved, and the body temperature was 36.8°C, but the symptoms of coughing were not relieved, leading us to speculate that this may be related to the continuous compression of the tumor. After 2 weeks of chemotherapy according to the aforementioned regimen, the blood routine was monitored, and the blood parameters were reduced (WBC: 1.17 × 10^−9^/L; red blood cell (RBC): 2.79 × 10^−12^/L; Hb: 68 g/L; PLT: 123 × 10^−9^/L), considering the possibility of bone marrow suppression after chemotherapy; thus, patients were given an infusion of RBCs to correct anemia and recombinant human granulocyte colony-stimulating factor to stimulate bone marrow hematopoiesis, and temporarily chemotherapy was stopped. On day 11, after the suspension of chemotherapy, the patient’s mental appearance improved compared to before, and the blood test showed that the blood routine recovered (WBC: 4.41 × 10^−9^/L; RBC: 4.05 × 10^−12^/L; Hb: 85 g/L; PLT: 553 × 10^−9^/L). Then, he started to continue the treatment according to the chemotherapy regimen of EP regimen (etoposide + cisplatin) and paclitaxel + ifosfamide + cisplatin + bleomycin chemotherapy regimen. During the treatment period, the patient’s blood routine was monitored at an average of 2 weeks to observe the patient’s tolerance to the chemical regimen, body temperature, and mental outlook and perform a full physical examination if necessary. After 4 months of treatment with the aforementioned chemotherapy regimen for six cycles, the patient underwent a whole-body PET/CT examination to evaluate the treatment effect. PET/CT showed that the tumor volume was not significantly reduced compared to before treatment (Figure 4), but no obvious systemic metastasis was observed. Hitherto, the patient has been followed up for 6 months, and every two courses of treatment, he will undergo a chest CT scan to assess the changes in tumor volume; also, he is still actively undergoing chemotherapy and is waiting for the opportunity to undergo surgical treatment.

**Table 1:** Laboratory examination and tumor marker test results

Index	Value	Unit	Annotation	Reference
WBC	12.13 × 10^−9^	/L	Up	(4–10) × 10^−9^
Hemoglobin	86.0	g/L	Down	110–160
Platelet	380 × 10^−9^	/L	Up	(100–300) × 10^−9^
Calcium	2.08	mmol/L	Down	2.25–2.27
Phosphorus	1.22	mmol/L	Down	1.30–1.62
Albumin	31.3	g/L	Down	35–51
NSE	57.41	ng/mL	Up	<17.00
CA199	681.40	U/mL	Up	<40.00
CA125	90.90	U/mL	Up	<35.00
AFP	2318.25	ng/mL	Up	<20.00
CYFRA21-1	2.62	ng/mL	Negative	<3.30
SCCA	13.40	ng/mL	Negative	<70.00
CEA	2.93	μg/L	Negative	<5.00

Notes: WBC = white blood cell; NSE = neuron-specific enolase; CA = carbohydrate antigen; AFP = alpha fetal protein; SCCA = squamous cell carcinoma antigen; CYFRA = cytokeratin fragment; CEA = carcino-embryonic antigen.

**Informed consent**: Both oral and written informed consent from the patient and his parents to make the patient’s details public were obtained.
**Ethical approval**: The research related to human use follows the principles of the Helsinki Declaration and has been approved by the Ethics Committee of the Affiliated Hospital of Zunyi Medical University.

### 2.2 Literature review

PubMed and Web of Science databases were searched, and English case reports and case series on mediastinal MGCT published before August 1, 2020, were retrieved. The following keywords (mediastinum OR mediastinal) AND (Mixed germ cell tumor OR MGCT OR mixed GCT) were used for retrieval. A total of eight full-text articles, including nine patients with mediastinal MGCT, were retrieved for the analysis. The first author and references for each case, as well as the patient’s age, gender, maximum tumor diameter, symptoms, CT image characteristics, follow-up results, and treatment, are presented in Table 2.

**Figure 5:**
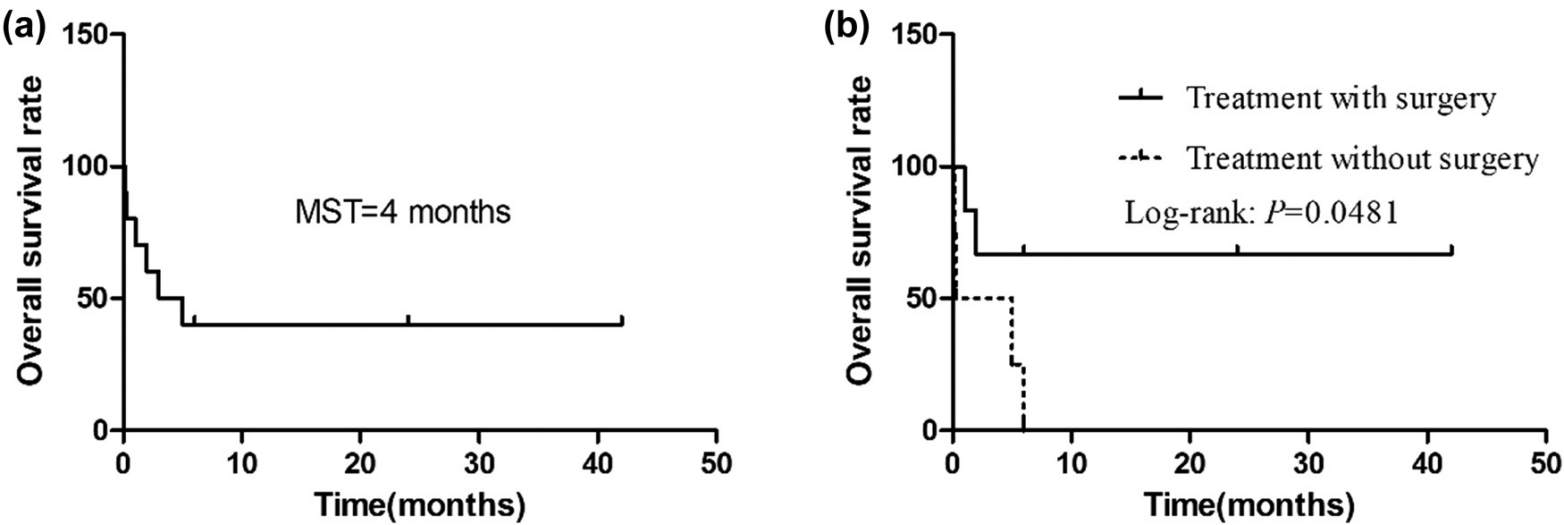
Kaplan–Meier OS. (a) OS in all cases. (b) OS by treatment. MST, median survival time.

Finally, only eight full-text articles of mediastinal MGCT patients were retrieved from the literature, indicating that MGCT is not common in mediastinum [11,12]. All the 10 patients (including our case) were males, aged 8.5–40 years, and the median age of the onset was 26 years. The tumor volume was large, and the maximum diameter line of all the eight patients was >10 cm (average: 15.8 cm). Moreover, nearly half (4/10) of our enrolled patients developed symptoms such as fever and sweating, which were not common in other mediastinal tumors. In the literature, the treatment of MGCT was mainly postoperative chemotherapy. When the lesion volume is large or distant metastasis occurs, patients only get the opportunity of chemotherapy. Overall, the prognosis of MGCT was poor, and the patient who received surgical resection had a better prognosis than those who received chemotherapy alone. The survival curves of the 10 enrolled patients are shown in Figure 5.

## 3 Discussion

The mediastinal MGCT might be related to the abnormal migration and malignant transformation of the primordial germ cells during embryogenesis, resulting in the production of *in vitro* germ cells or the failure of the original germ cells to complete the normal migration from the urogenital ridge to the gonad ridge during embryonic development [13]. The extragonadal germ cell tumors account for about 10% of all germ cell tumors, occurring mainly in the central axis of the body, such as retroperitoneum, mediastinum, and brain [8,14]. The mediastinal MGCT accounts for about 20% of all mediastinal germ cell tumors, and mediastinal MGCT accounts for <0.5% of mediastinal tumors, with a relatively low incidence [8]. A review of the published literature concluded that the development of extragonadal germinomas is strongly associated with the Klinefelter syndrome, which serves as an indicator of mediastinal germinomas in young patients [15]. The clinical manifestations of the mediastinal MGCT were similar to those of other mediastinal tumors: most patients may have chest and back pain, chest discomfort, cough, dyspnea, palpitations, and other symptoms due to tumor oppression on adjacent tissues. The values of serum AFP, human chorionic gonadotropin (HCG), and lactate dehydrogenase (LDH) were increased in MGCT patients. In our enrolled patients, more than half of the patients showed positive expression of AFP and HCG. Needle aspiration biopsy is a critical examination method for the diagnosis of mediastinal MGCT, but recently, Sakane et al. [6] reported a case of mediastinal MGCT patient with diffuse lung metastasis caused by puncture biopsy. Therefore, if noninvasive imaging tests such as CT could characterize tumor tissue, the incidence of such events could be reduced. In the current group of patients, the CT data of eight patients (8/9) with mediastinal MGCT were described. The CT findings of all patients were heterogeneous due to cystic necrosis of the tumor, including five cases of hemorrhage and four cases of calcification. In addition, contrast-enhanced CT (CE–CT) examination was performed in this patient, which showed mild and moderate nonuniform enhancement, and low-density nonenhanced necrotic areas were observed in the tumor, which was consistent with the CT findings of mediastinal MGCT reported in the literature [16].

Based on the aforementioned characteristics, mediastinal MGCT needs to be differentiated from mediastinal teratoma, isolated fibroma, neurogenic tumor, lymphoma, and metastatic tumors. The CT manifestations of teratomas are well-defined cystic solid masses. Mature teratomas may be easily diagnosed due to their inclusion of mixed densities of adipose tissue, calcification, and bone. A solitary fibroma is usually manifested as a soft tissue mass with uniform density, but when the tumor is large, cystic degeneration and necrosis may occur, presenting an uneven density. In addition, the CE–CT showed obvious enhancement of lesions, and enhanced tumor vascular shadows were relatively specific. Neurogenic tumors appeared as a round or spindle-shaped soft tissue mass in the mediastinal thoracic cavity. Various pathological types may have different imaging findings, the density of benign lesions is relatively uniform, the malignant lesions are low-density cystic necrosis and/or high-density calcification lesions, and the lesions exhibit heterogeneous enhancement on CE–CT. Mediastinal lymphoma manifests as an irregular soft tissue mass in the mediastinum on CT, and multiple enlarged lymph nodes can be seen in the lesion with uniform density but with rare necrosis and calcification. The lesion surrounds the growth of large blood vessels and shows mild to moderate enhancement on CE–CT. Metastatic tumors, with a history of primary tumors, are mostly multiple foci, necrosis is common, and uneven enhancement on CE–CT could be identified on PET/CT.

**Table 2:** Clinical information of 10 patients (including our case) with mediastinal MGCT

First author/nation	Sex/age	MD (cm)	CT	HCG/AFP	Pathological components	History	Treatment	Follow-up (months)
			D/CN/H/Ca					
Pradhan/India [3]	M/40	18.0	I/+/+/−	−/−	YST (70%) + MT (30%)	Fever + cough + pain	C	5 (1)
Yuri/Japan [4]	M/19	12.6	−	+/−	CC + MT	Pain + fever + dyspnea	C	0.2 (1)
Taccagni/Italy [5]	M/27	15.0	I/+/−/+	−/−	EC + IT	Cough + pain	S + C	2 (1)
Sakane/Japan [6]	M/27	11.0	I/+/−/+	+/+	CC + YST + EC + Se	Back pain	C	0.3 (1)
Ruan/China [7]	M/28	12.0	I/+/+/−	+/+	CC (90%) + IT (10%)	Palpitation + hydrosis + tachypnea	S + C	24 (0)
Fritzsche/Switzerland [8]	M/26	21.0	I/+/−/+	+/+	Se + IT	Cough + dyspnea	S + C	1 (1)
Bakshi/India [9]	M/28	12.0	I/+/+/−	−/−	MT + Se	Cough + chest discomfort	S + C	6 (0)
Bakshi/India [9]	M/17	15.6	I/+/−/−	−/−	YST + MT	Chest pain	S + C + R	6 (0)
Völkl/Germany [10]	M/8.5	5.0	I/+/−/+	+/+	CC + YST + EC + Se	Klinefelter syndrome	S + C	42 (0)
Present case	M/12	25.0	I/+/−/+	−/+	YST + IT	Fever + cough	C	6 (0)

Notes: M = male; MD = maximum diameter; CT = computed tomography; D = density; I = inhomogeneous; CN = cystic necrosis; H = hemorrhage; Ca = calcification; YST = yolk sac tumor; MT = mature teratoma; CC = choriocarcinoma; EC = embryonal carcinoma; Se = seminoma; IT = immature teratoma; C = chemotherapy; S = surgery; R = radiotherapy; “+” means positive; “−” represents negative or not performed. “0” indicates that the patient has not reached the terminal event, and “1” indicates that the terminal event has been reached, “terminal event” was defined as the patient’s death from the disease (mediastinal mixed germ cell tumor) or related complications during the last follow-up period.

Strikingly, like most tumors, histopathological examination is crucial for the accurate diagnosis of MGCT, but the disadvantage of preoperative biopsy is that only a little tissue was obtained, which might lead to missed or incomplete diagnosis of tumor histopathological components [11,17]. However, if sufficient tissue was obtained, it might increase the risk of tumor metastasis (6). Therefore, we speculated that preoperative biopsy is unnecessary if the tumor is small and can be surgically removed. However, if the tumor is large, not surrounded by large blood vessels, is close to the body surface, and cannot be removed surgically, a tissue biopsy under the guidance of CT is feasible because an accurate pathological diagnosis can provide the patient with a detailed chemotherapy regimen.

Complete surgical resection of tumor tissue is a key factor in the prognosis of mediastinal MGCT. However, the majority of the patients have lost the opportunity of surgical treatment due to large tumor volume, the embedding of large peripheral vessels, and distant metastasis at the time of diagnosis. In these patients, chemotherapy regimens of EP (etoposide plus cisplatin) and paclitaxel plus ifosfamide plus cisplatin are administered. Almost half (4/9) of our patients received chemotherapy alone, and even after surgery, some patients [5,8] died of respiratory failure within a short period. Therefore, the prognosis of the patients with mediastinal MGCT was extremely poor. The average survival time of the 10 patients in this group was only 3 months.

## 4 Conclusion

In a nutshell, mediastinal MGCT has a low incidence rate and some difficulties in diagnosis. A large soft tissue mass was detected in the mediastinum with low-density necrosis and high-density calcification. The contrast agent dynamic enhanced scan showed mild and moderate progressive enhancement with a specific value for the diagnosis of mediastinal MGCT. MGCT has a high degree of malignancy and poor prognosis. Therefore, an in-depth understanding of the clinical and imaging knowledge of this disease is the key to early diagnosis, early intervention treatment, and improvement of prognosis.

## Abbreviations

MGCT: mixed germ cell tumor
MIP: maximum intensity projection
CT: computed tomography
FDG: fluorodeoxyglucose
PET/CT: positron emission tomography/computed tomography
